# Page Kidney Following Ruptured Renal Angiomyolipoma Managed With Selective Embolization: A Case Report

**DOI:** 10.7759/cureus.108976

**Published:** 2026-05-16

**Authors:** Rayan Alataa, Katherine Rivero, Kriti Gupta, Hanasoge Girishkumar, Charbel Ishak

**Affiliations:** 1 Internal Medicine, BronxCare Health System, Bronx, USA; 2 General Surgery, BronxCare Health System, Bronx, USA; 3 Surgery, BronxCare Health System, Bronx, USA; 4 Interventional Radiology, Bronxcare Health System, Affiliate of Icahn School of Medicine at Mount Sinai, Bronx, USA

**Keywords:** page kidney, renal angiomyolipoma, spontaneous perinephric hematoma, transarterial embolization, wunderlich syndrome

## Abstract

Spontaneous perinephric hematoma (SPH), or Wunderlich syndrome, is a rare but potentially life-threatening emergency most often caused by the rupture of a renal angiomyolipoma (AML).

A 60-year-old woman presented with sudden-onset right flank pain and anemia in the absence of trauma. Imaging revealed a large right perinephric hematoma secondary to a ruptured renal AML with giant aneurysms and pseudoaneurysms. Despite the extensive hemorrhage, she remained hemodynamically stable and underwent urgent selective transarterial embolization. Post-embolization imaging confirmed complete hemostasis with preservation of renal parenchyma. A compressive perinephric hematoma resulted in transient Page kidney physiology, which was managed conservatively with blood pressure control.

This case illustrates several uncommon features: hemodynamic stability despite massive hemorrhage, concurrent giant aneurysms and pseudoaneurysms, and the development of Page kidney. It underscores the diagnostic utility of contrast-enhanced CT and the increasing role of selective endovascular therapy as a nephron-sparing alternative to nephrectomy. Emerging evidence suggests that vascular complexity within AMLs, particularly giant aneurysms and pseudoaneurysms, may be more predictive of rupture than tumor size alone.

Prompt imaging, multidisciplinary coordination, and minimally invasive transarterial embolization can yield excellent outcomes in ruptured AML with complex vascular anatomy. Early angiographic evaluation should be considered in symptomatic or high-risk AMLs, with close monitoring for Page kidney to optimize long-term renal preservation.

## Introduction

Spontaneous perinephric hematoma (SPH), also referred to as Wunderlich syndrome, is an uncommon but potentially fatal urological emergency characterized by non-traumatic renal or perirenal hemorrhage contained beneath Gerota’s fascia [1]. The presentation is often dramatic but nonspecific, ranging from mild flank discomfort to life-threatening hemorrhagic shock. Etiologies vary and include renal neoplasms, vascular lesions, connective tissue disorders, coagulopathies, infection, and inflammatory diseases [2]. Among the underlying causes, renal angiomyolipoma (AML), a benign mesenchymal tumor composed of adipose tissue, smooth muscle, and dysplastic vasculature, represents the most frequent benign source of spontaneous renal bleeding and ranks second overall, after renal cell carcinoma [3].

Although most AMLs are incidentally detected and clinically silent, lesions exceeding 4 cm or harboring intralesional aneurysms possess a markedly higher risk of rupture due to structural vascular fragility and hemodynamic stress [4,5]. Additional rupture-promoting factors include pregnancy, hormonal fluctuations, trauma, and anticoagulation therapy. While the classic Lenk’s triad, the three hallmark symptoms of retroperitoneal hemorrhage consisting of flank pain, a palpable abdominal mass, and hypovolemic shock, has been traditionally described, it is encountered in fewer than 20% of cases, making early diagnosis challenging [1,6]. Timely imaging plays a critical role. Contrast-enhanced computed tomography (CT) remains the diagnostic modality of choice given its ability to accurately delineate hematoma extent, identify fat-containing renal lesions, and evaluate associated vascular abnormalities [6].

Historically, emergent nephrectomy served as the standard of care for hemodynamically unstable patients or in cases suspected of malignancy. However, the paradigm has shifted substantially with the increasing adoption of selective arterial embolization (SAE), now recognized as a safe, kidney-preserving therapeutic alternative capable of rapidly controlling hemorrhage while maintaining renal function [7-9].

We describe a rare case of massive SPH secondary to a ruptured AML with giant aneurysms and pseudoaneurysms. The case further illustrates transient Page kidney physiology, a condition in which external compression of the kidney by a perinephric hematoma impairs renal perfusion, triggering renin-angiotensin-mediated secondary hypertension. This case also underscores the importance of recognizing its hemodynamic consequences. The report adds to the growing literature supporting multidisciplinary, minimally invasive management strategies and highlights the prognostic implications of vascular morphology in AML rupture risk assessment. The rare convergence of massive hemorrhage with hemodynamic stability, complex giant pseudoaneurysms, and the subsequent development of Page kidney is considered rare.

## Case presentation

A 60-year-old woman with no significant past medical history presented to the emergency department with right flank pain of 5 days' duration, which had acutely worsened over the preceding 24 hours. She described the pain as moderate (6/10), gradual in onset, non-radiating, and not preceded by trauma. She denied gross hematuria, dysuria, fever, or chills. On presentation, her vital signs were stable. Abdominal examination revealed a soft, mildly distended abdomen with mild right flank tenderness.

Laboratory evaluation demonstrated leukocytosis, marked microcytic anemia (hemoglobin 6.7 g/dL), and significant thrombocytosis (platelet count 518 k/uL). The basic metabolic panel, liver function tests, and coagulation profile were within normal limits 9 (Table 1).

**Table 1:** Laboratory findings on admission APTT: Activated Partial Thromboplastin Time; HGB: Hemoglobin; INR: International Normalized Ratio; PT: Prothrombin Time; RBC: Red Blood Cell; WBC: White Blood Cell

Test	Result	Normal Range
HGB	6.7	12-16 g/dl
Platelet	518	150-400 k/ul
WBC Count	12	4.8-10.8 k/ul
RBC	2.88	4.50-5.90 M/ul
Potassium, Serum	4.4	3.5-5 mEq/L
Sodium, Serum	140	135-145 mEq/L
Chloride, Serum	102	98-108 mEq/L
Calcium, Total Serum	9.9	8.5-10.5 mg/dL
Creatinine, Serum	0.9	0.5-1.5 mg/dL
Blood Urea Nitrogen, Serum	13.0	8-26 mg/dL
PT	12.1	10.4-15.7 sec
APTT	37.6	28-42.9 sec
INR	0.99	0.85-1.29
Bilirubin, Serum Total	0.4	0.2-1.2 mg/dL
Albumin, Serum	3.6	3.4-4.8 g/dL
Aspartate Transaminase, Serum	28	9-48 unit/L
Alkaline Phosphatase, Serum	119	53-128 unit/L
Bilirubin, Serum Direct - Conjugated	<0.2	0.0-0.3 mg/dL
Alanine Aminotransferase, Serum	21	5-40 unit/L

**Figure 1:**
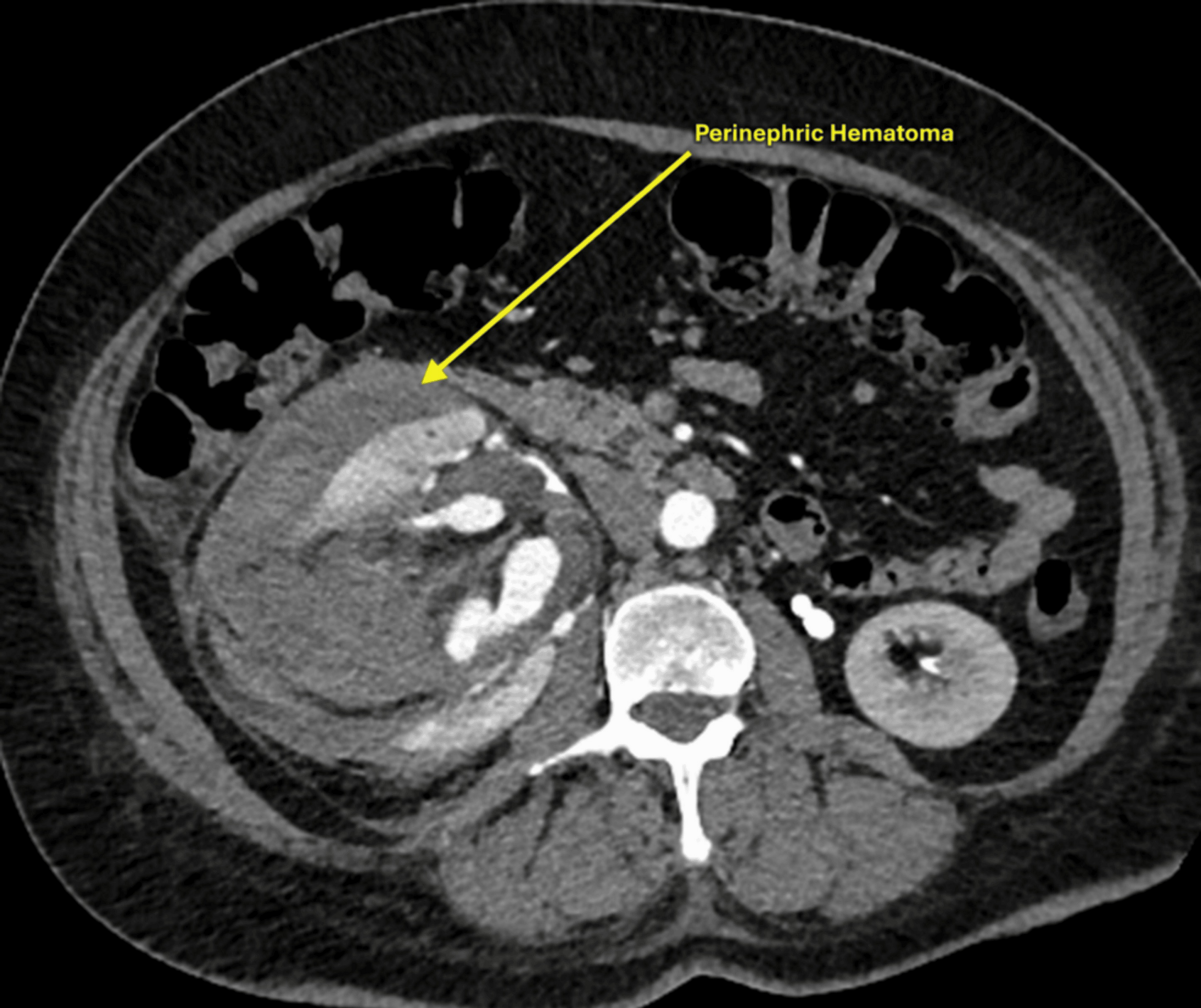
Axial-enhanced CT images demonstrating a large, hyperdense perinephric hematoma (yellow arrow) encasing and compressing the right kidney The hematoma caused significant enlargement of the right renal fossa.

**Figure 2:**
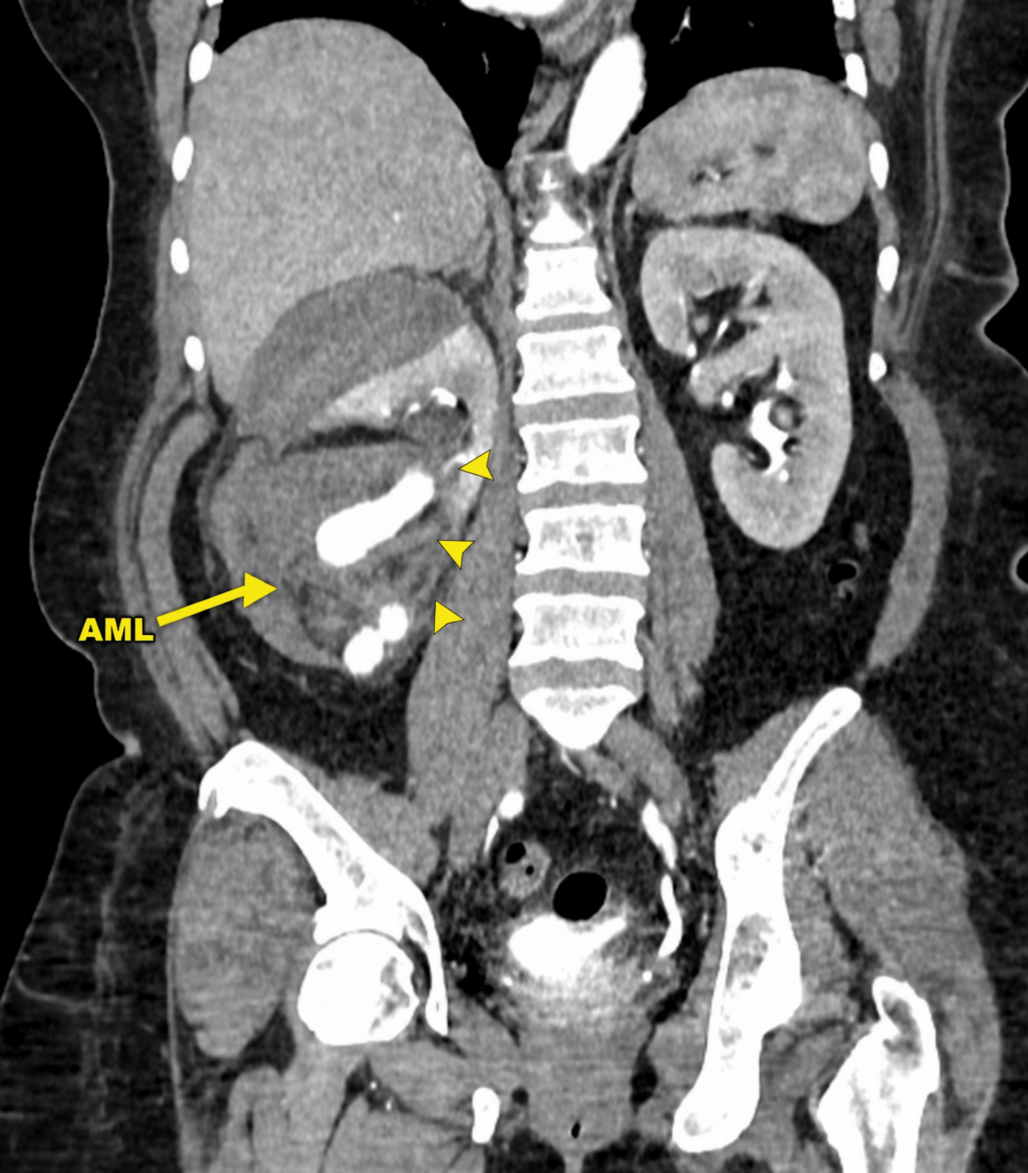
Coronal CT angiography images confirming the 12 x 14 cm right perirenal hematoma A heterogeneous, fat-containing mass consistent with an angiomyolipoma (arrow) is identified at the inferior pole of the right kidney, with associated giant aneurysms and pseudoaneurysms (arrowheads). AML: Angiomyolipoma

On day one of admission, the initial contrast-enhanced CT of the abdomen and pelvis (CTAP) demonstrated a markedly enlarged right kidney surrounded by a large, acute perinephric hematoma (Figure 1). To better characterize the underlying cause, a subsequent CT angiography (CTA) of the chest, abdomen, and pelvis was performed. The CTA confirmed the presence of a large, 12 × 14 cm right perirenal hematoma, which was exerting a significant mass effect on the adjacent renal parenchyma. The source of the hemorrhage was identified as a heterogeneous, fat-containing mass at the inferior pole of the right kidney, a finding consistent with AML. Associated with this mass were multiple giant aneurysms, pseudoaneurysms, and markedly dilated vasculature (Figure 2). The CTA of the chest also revealed an incidental pulmonary embolism.

**Figure 3:**
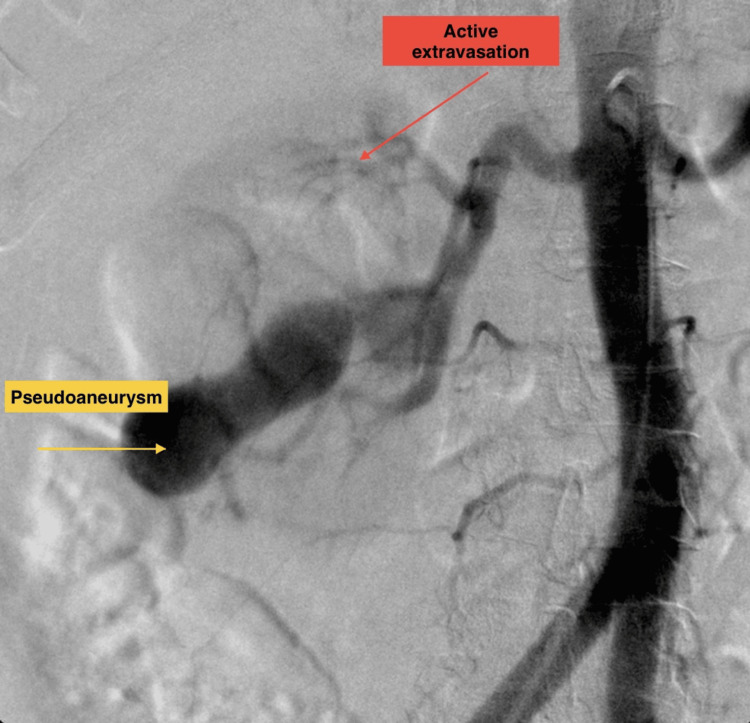
Digital subtraction angiography (DSA) image of the right renal artery demonstrating a hypervascular mass at the lower pole with multiple giant aneurysms and pseudoaneurysms Active contrast extravasation is visible, indicating ongoing hemorrhage from the ruptured angiomyolipoma.

**Figure 4:**
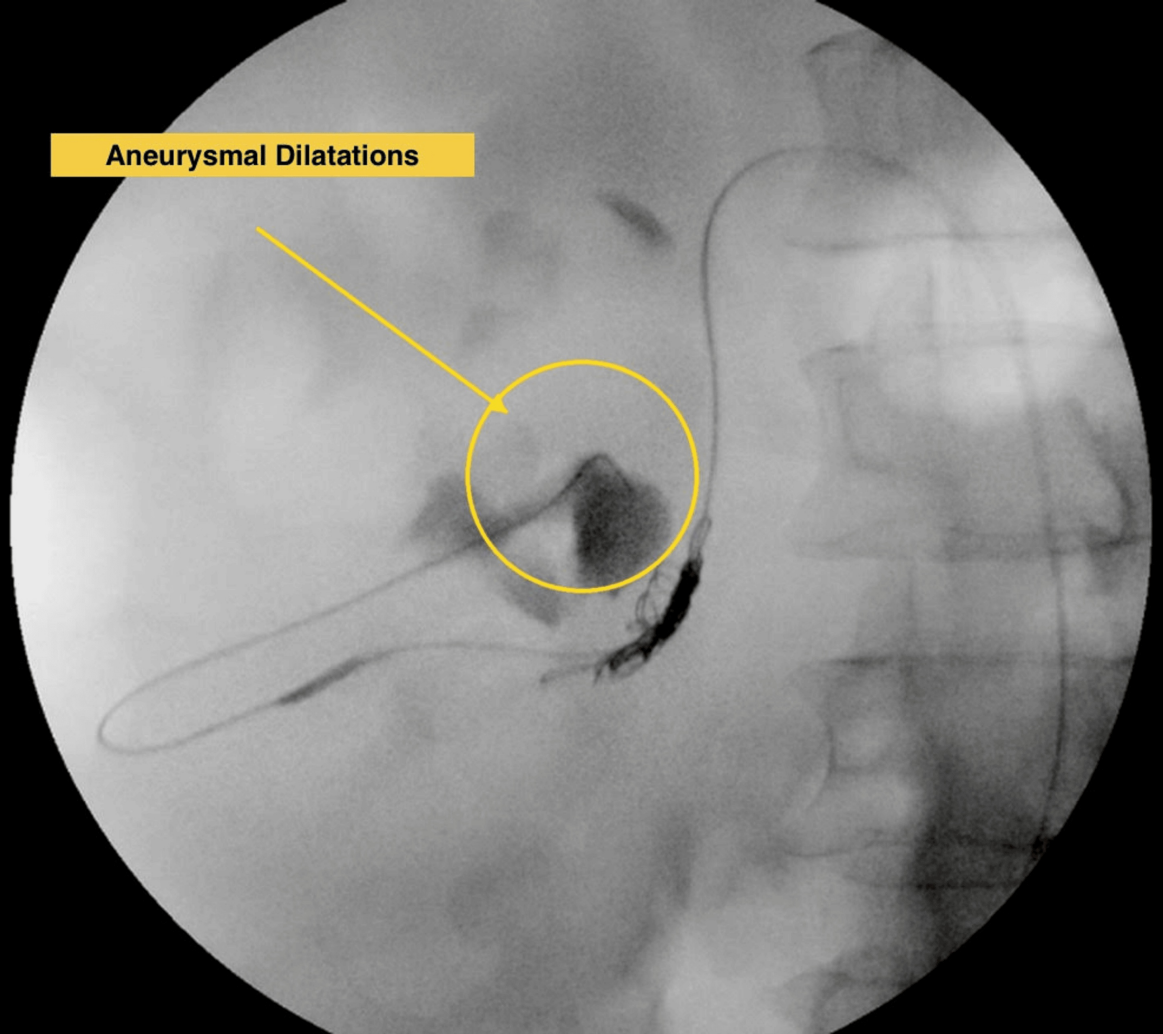
Selective right renal angiography image showing the complex vascular architecture of the angiomyolipoma, with tortuous, dilated feeding vessels and multiple aneurysmal dilatations prior to embolization

**Figure 5:**
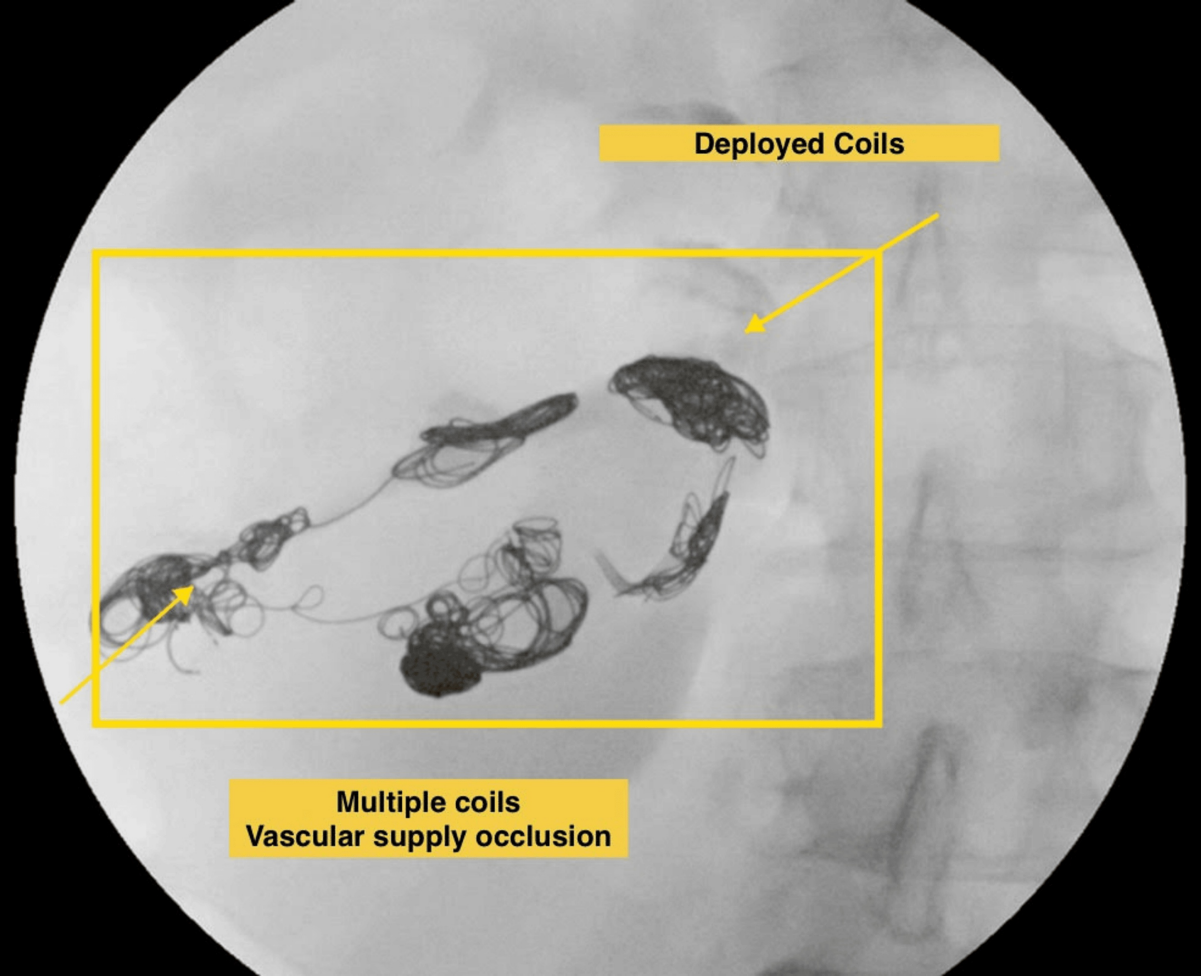
Fluoroscopic image during super-selective transarterial embolization showing deployment of multiple coils within the feeding vessels of the angiomyolipoma The coils are strategically placed to occlude vascular supply to the tumor.

The patient was admitted to the intensive care unit. She received 2 units of packed red blood cells, with hemoglobin stabilizing at 8.5 g/dL. Interventional radiology performed selective and super-selective right renal angiography via right common femoral artery access. Angiography demonstrated active extravasation from a ruptured lower-pole AML with associated giant aneurysms and pseudoaneurysms (Figure 3). Super-selective transarterial embolization of the feeding vessels was performed using coils, followed by Gelfoam slurry (Figure 4, Figure 5). Post-embolization angiography showed complete cessation of active bleeding. The right lower renal cortex demonstrated poor perfusion and absent contrast excretion compared with the contralateral kidney.

**Figure 6:**
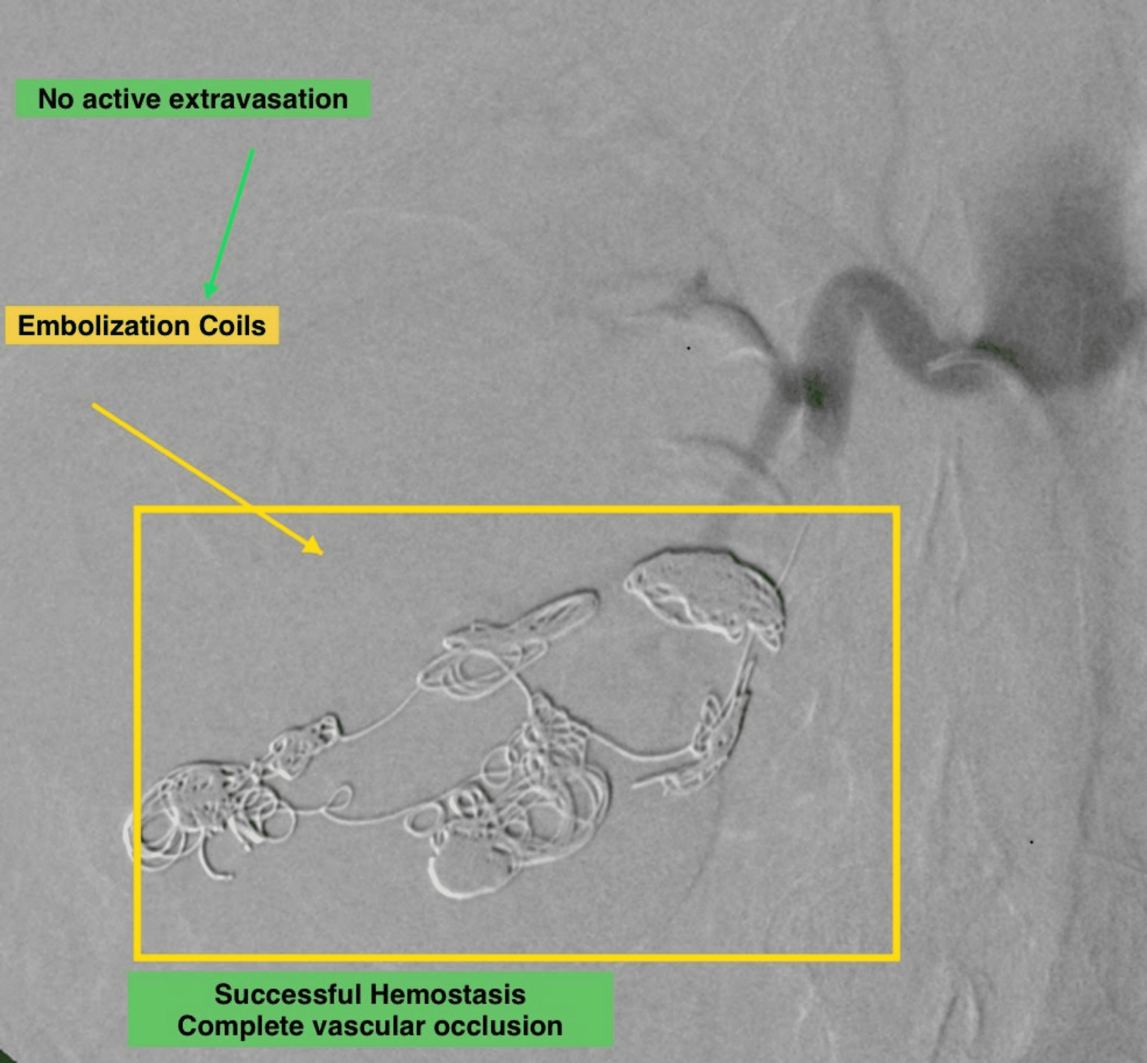
Post-embolization angiography image demonstrating complete cessation of blood flow to the angiomyolipoma and absence of active contrast extravasation Multiple embolization coils are visible within the previously hypervascular tumor bed, confirming successful hemostasis.

Post-embolization, the patient remained hemodynamically stable with significant improvement in flank pain (Figure 6). Therapeutic anticoagulation for her pulmonary embolism was initiated once stability was confirmed, without evidence of recurrent bleeding. However, by hospital day 3, she developed new-onset hypertension with blood pressure readings peaking at 175/105 mmHg, consistent with Page kidney physiology secondary to the compressive perinephric hematoma impairing renal perfusion and activating the renin-angiotensin-aldosterone system. Antihypertensive therapy was initiated with amlodipine 5 mg daily, with subsequent improvement in blood pressure to 138/88 mmHg by hospital day 5. She was monitored for post-embolization syndrome and had an otherwise smooth inpatient course.

**Figure 7:**
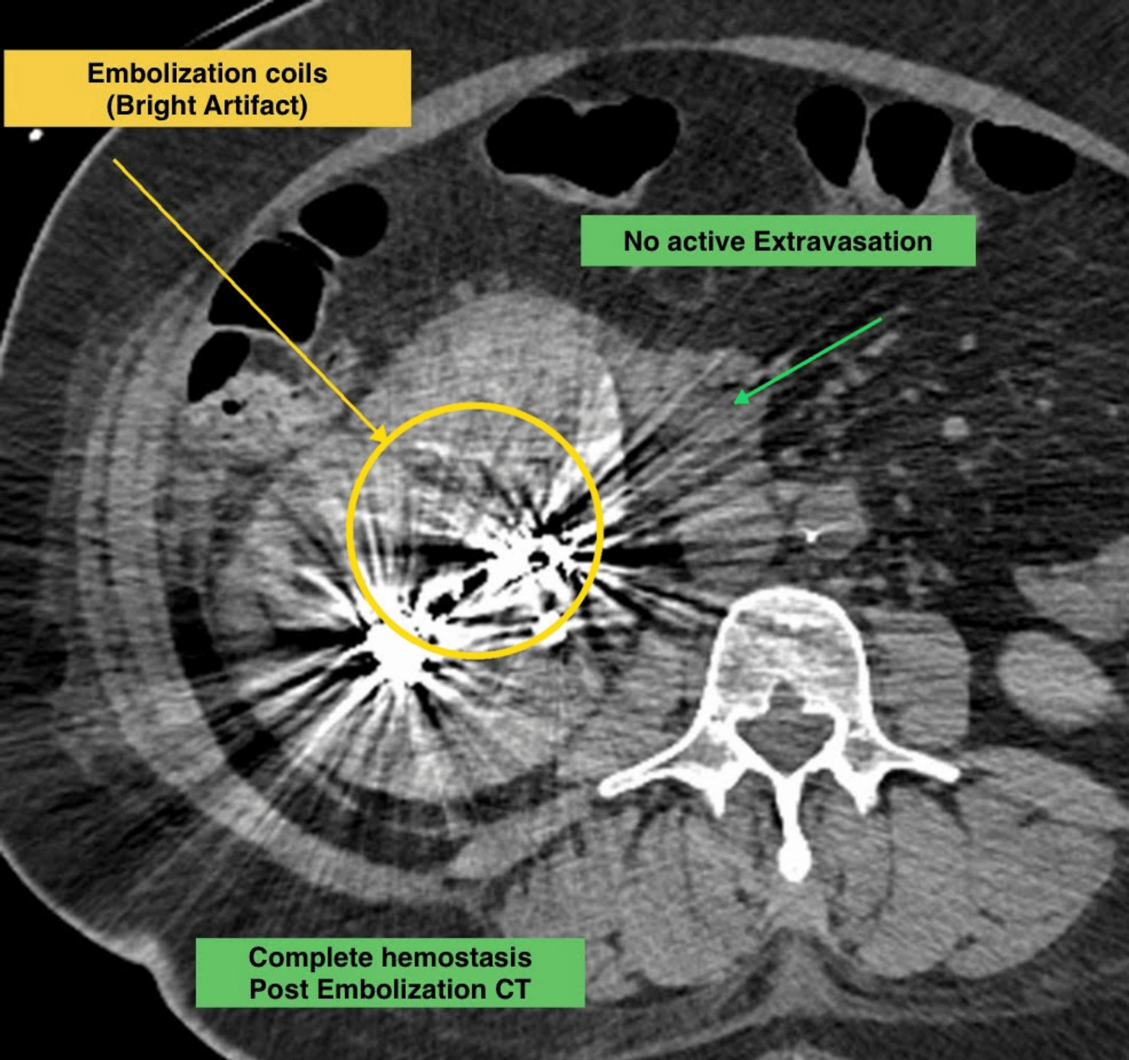
Axial post-embolization contrast-enhanced CT image showing multiple embolization coils (bright artifacts) within the right kidney at the site of the treated angiomyolipoma No active contrast extravasation is identified, indicating complete hemostasis.

Follow-up CT after embolization confirmed cessation of bleeding and preservation of the majority of the renal parenchyma, with serum creatinine remaining stable at 1.1 mg/dL, confirming renal functional preservation (Figure 7). The patient was discharged on hospital day 7 with well-controlled blood pressure of 128/82 mmHg on amlodipine 5 mg daily. Repeat outpatient imaging demonstrated progressive resorption of the hematoma and mild residual renal compression. Blood pressure normalized over the following weeks with progressive hematoma resorption, and antihypertensive therapy was subsequently tapered and discontinued.

## Discussion

Renal angiomyolipoma is well-established as the leading benign etiology of Wunderlich syndrome, most commonly affecting middle-aged females and occasionally presenting with catastrophic hemorrhage [2,4]. The imaging characteristics in this patient, a fat-containing renal lesion with aneurysmal vascular changes, aligned strongly with previously described AML presentations, supporting AML rupture as the primary hemorrhagic source [3,4]. While AML size has traditionally guided risk stratification, emerging evidence suggests that aneurysm formation (>5 mm) and vascular complexity may be stronger predictors of rupture. Our case is consistent with this observation, though definitive conclusions cannot be drawn from a single case report [4,5].

Massive spontaneous perirenal hemorrhage events remain relatively uncommon in the published literature, and when they do occur, they often involve hemodynamic instability, anticoagulation, or systemic comorbidities [10-12]. Notably, our case demonstrates hemodynamic stability despite a large hematoma, an infrequent finding that permitted structured imaging evaluation and non-surgical intervention, reinforcing that not all ruptured AMLs mandate emergent nephrectomy [1,2]. The coincidental discovery of pulmonary embolism also adds clinical interest, as concurrent spontaneous perirenal hemorrhage and venous thromboembolism have rarely been reported together and may reflect transient inflammatory or hematologic activation [11,12].

Diagnostic imaging remains pivotal, with contrast-enhanced CT and CT angiography serving as cornerstone modalities to characterize hematoma volume, active bleeding, tumor composition, and underlying vascular networks, including giant aneurysms and pseudoaneurysms [6,13,14]. MRI remains valuable in cases where AMLs lack macroscopic fat or when vascular identification is inconclusive [6].

The therapeutic landscape has evolved significantly. Whereas nephrectomy historically dominated hemorrhage control, increasing data support selective or super-selective embolization as first-line therapy due to high success rates, renal preservation, shortened hospital stays, and the added benefit of tumor devascularization and size regression over time [6-9]. Our patient's excellent response, complete hemostasis, parenchymal preservation confirmed by stable creatinine at 1.1 mg/dL, and rapid symptom improvement mirror the outcomes of recent interventional radiology series [8,9]. Nevertheless, embolization failure, ongoing hemodynamic instability, or suspicion of malignancy remain indications for surgical intervention, and nephrectomy retains a role in salvage management [15-17]. Conservative management may be appropriate in select, self-limited hemorrhages but requires cautious clinical and radiologic follow-up [18].

A notable clinical learning point demonstrated in our case is the development of Page kidney, a condition in which a compressive perirenal hematoma impairs renal perfusion, activating the renin-angiotensin-aldosterone system and driving secondary hypertension. In our patient, blood pressure rose to 175/105 mmHg by hospital day 3, prompting initiation of amlodipine 5 mg daily, with subsequent improvement to 128/82 mmHg at discharge. Blood pressure normalized over the following weeks as the hematoma resorbed, confirming the transient nature of this phenomenon. Early recognition and prompt antihypertensive management prevented long-term sequelae and exemplified the importance of vigilant hemodynamic monitoring following perirenal hemorrhage.

Taken together, this case supports several emerging principles in AML management. Vascular morphology, particularly the presence of giant aneurysms and pseudoaneurysms, may surpass tumor size as a predictor of rupture risk, though prospective data are needed to validate this observation. Multidisciplinary early imaging and endovascular control appear to provide durable, kidney-sparing outcomes. Prophylactic transarterial embolization may warrant consideration in AMLs with high-risk vascular features as a topic for future investigation [9]. Finally, awareness of Page kidney physiology is essential for blood pressure management and long-term renal preservation following perirenal hemorrhage.

## Conclusions

This case highlights the critical importance of recognizing complex vascular architecture, specifically giant aneurysms and pseudoaneurysms, as a primary driver of rupture risk in renal angiomyolipomas, independent of absolute tumor size. Furthermore, it demonstrates that even in the setting of massive spontaneous perinephric hemorrhage (Wunderlich syndrome), hemodynamic stability can be maintained, allowing for a structured diagnostic approach with contrast-enhanced computed tomography.

The successful management of this patient underscores the paradigm shift toward nephron-sparing interventions. Super-selective transarterial embolization proved to be a highly effective, minimally invasive first-line therapy that rapidly achieved hemostasis while preserving the majority of the renal parenchyma. Finally, clinicians must remain vigilant for the delayed development of Page kidney physiology following large subcapsular or perinephric hematomas. Early recognition of renin-angiotensin-mediated hypertension and prompt initiation of conservative medical management are essential to optimizing long-term renal function and preventing irreversible ischemic injury.
